# Discriminatory power of trabeculectomy bleb internal reflectivity and morphology in surgical success using anterior segment optical coherence tomography

**DOI:** 10.1186/s12886-024-03770-6

**Published:** 2025-01-30

**Authors:** Jeremy C.K. Tan, Matthew Roney, Matteo Posarelli, Abdus Samad Ansari, Mark Batterbury, Neeru A. Vallabh

**Affiliations:** 1 St Paul’s Eye Unit, Royal Liverpool University Hospital, Liverpool, UK; 2 Faculty of Medicine and Health, University of New South Wales, Kensington, NSW Australia; 3 Glaucoma service, Moorfields Eye Hospital NHS Foundation Trust, London, UK; 4 Department of Eye and Vision Sciences, Institute of Life Course and Medical Sciences, University of Liverpool, Liverpool, UK

**Keywords:** Glaucoma surgery, Trabeculectomy, Mitomycin-C, Scleral flap, Sclerostomy, Surgical technique, Surgical success

## Abstract

**Background:**

The post-operative evaluation of trabeculectomy blebs has traditionally relied on subjective clinical grading systems performed at the slit-lamp. This study explores the use of swept source anterior-segment optical coherence tomography (AS-OCT) to objectively measure bleb internal reflectivity and morphology, and to distinguish blebs with surgical success vs. failure.

**Methods:**

Cross-sectional study of patients with glaucoma who had undergone trabeculectomy at least one year prior. Swept source AS-OCT was used to capture filtering blebs in the sagittal plane. Standardised regions of interests on the sagittal plane were segmented, and pixel intensity values and bleb height were measured. Receiver operating characteristic curves were used to examine the discriminatory ability of pixel intensity values and bleb morphology to classify blebs with surgical success or failure.

**Results:**

100 eyes of 65 patients were included, with a median post-operative follow up of 7.0 years (IQR 3.2–16 years). The proportion of complete success, qualified success and failure was 45%, 33%, and 22% respectively. The maximum bleb height was significantly greater in the blebs with complete success (1.74 vs. 1.25 vs. 1.23 mm in CS vs. QS vs. F, one-way ANOVA, *p* < 0.0001). Mean pixel intensity was significantly lower in blebs with complete success (150.8 vs. 157.4 vs. 167.4 in CS vs. QS vs. F, *p* = 0.0001). Bleb intensity standard deviation (AUC 0.81), maximal bleb height (AUC 0.76), mean pixel intensity (AUC 0.75) and minimum pixel intensity (AUC 0.75) offered the best discrimination between surgical success and failure.

**Conclusions:**

Swept-source AS-OCT can be used to quantify bleb internal reflectivity and morphology, which can be used to distinguish between well vs. poorly functioning blebs. These parameters may assist surgeons in the objective evaluation of post-operative bleb outcomes.

## Introduction

Glaucoma is the second most common cause of vision loss, with approximately 57.5 million individuals globally believed to be impacted by the disease, a number expected to increase to 111.8 million by 2040 [1]. Currently the reduction of intraocular pressure (IOP) is the only known modifiable risk factor for the treatment of glaucoma [2]. Trabeculectomy is the gold standard in Glaucoma filtration surgery (GFS), which is performed to achieve greater intraocular pressure reduction or when other earlier interventions like topical medications fail to halt glaucoma progression. In trabeculectomy surgery, a permanent drainage outflow channel is created for aqueous egress from the anterior chamber to the sub-Tenon’s space [3]. Post-operative fibrosis and scarring of the sub-tenon’s space causes restriction of aqueous flow and overtime leads to failure. The long-term failure rate was reported to be 35% and 46.9% at 5-years of follow up in the Primary Tube versus Trabeculectomy study [4] and the Tube versus Trabeculectomy study [5] respectively.

The post-operative evaluation of GFS filtering blebs has traditionally relied on clinical grading systems performed at the slit-lamp such as the Indiana Bleb Appearance Grading Scale (IBAGS) and the Moorfields Bleb Grading System (MBGS), which document factors associated with surgical success such as bleb area, height and vascularity [6]. These factors can be used to guide pharmacological and/or surgical intervention in the early post-operative period, to decrease the risk of bleb fibrosis and failure. Variability in consensus and differing levels of interobserver agreement using these indices can however be marked and are influenced by the experience of the observer [7]. Subsequent studies have evaluated the microstructure of filtering blebs using non-contact imaging techniques such as Anterior Segment Optical Coherence Tomography (AS-OCT) and Ultrasound Biomicroscopy [6]. These modalities can provide quantitative data on the internal structure of blebs such as bleb wall thickness and intensity presence of microcysts and measurements of the internal ostium, bleb cavity and sub-flap space [6, 8–11], however measurements are often obtained manually.

The purpose of this study was to evaluate the use of swept-source AS-OCT to objectively evaluate bleb internal pixel intensity and morphology, and the discriminatory ability of these values in classifying patients with surgical success or failure.

## Methods

This was a cross-sectional study conducted at the St Paul’s Eye Unit, Royal Liverpool University Hospital, a tertiary referral eye unit in Liverpool, United Kingdom. The study had the approval of the clinical governance department of the Royal Liverpool University Hospital Trust (ID: 11783) with waiver of consent due to the retrospective nature of the study. The study adhered to the tenets of the Declaration of Helsinki. Study reporting was done in accordance to SQUIRE guidelines [12]. Consecutive patients attending the glaucoma clinics of the St Paul’s Eye Unit between December 2022 and March 2023 were recruited. The inclusion criteria were adult patients with a diagnosis of primary open angle glaucoma, pseudoexfoliative glaucoma, pigment dispersion syndrome glaucoma, or primary angle closure glaucoma, who had undergone trabeculectomy surgery with or without intraoperative mitomycin-C (MMC) at least 12 months prior to the index visit. Revision of primary trabeculectomy procedures were excluded.

### Trabeculectomy surgical technique

All trabeculectomy surgeries were performed by consultants and clinical fellows affiliated with the St Paul’s Eye Unit during this time period. The surgeries shared the following common steps—creation of a superior fornix-based conjunctival incision and peritomy, creation of a half-thickness rectangular scleral flap, punch sclerostomy, peripheral iridectomy, suturing of scleral flap with releasable/interrupted sutures, and conjunctival closure. Intraoperative mitomycin-C was used in the majority of cases and was performed following the conjunctival peritomy.

### Definitions of surgical success

The surgical outcome of each trabeculectomy case at the index visit was classified into complete success (CS; IOP ≤ 18mmHg with no medications), qualified success (QS; IOP ≤ 18 with medications) and failure (F; IOP > 18mmHg), as per the World Glaucoma Association consensus on definitions of success 2018 [13]. The cross-sectional nature of the study with long follow-up meant the pre-operative IOP was unknown in a large proportion of the patients, therefore we could not include a 20% reduction in baseline IOP as an additional criteria in defining surgical success.

### Anterior-segment optical coherence tomography imaging of filtering bleb

**Fig. 1:**
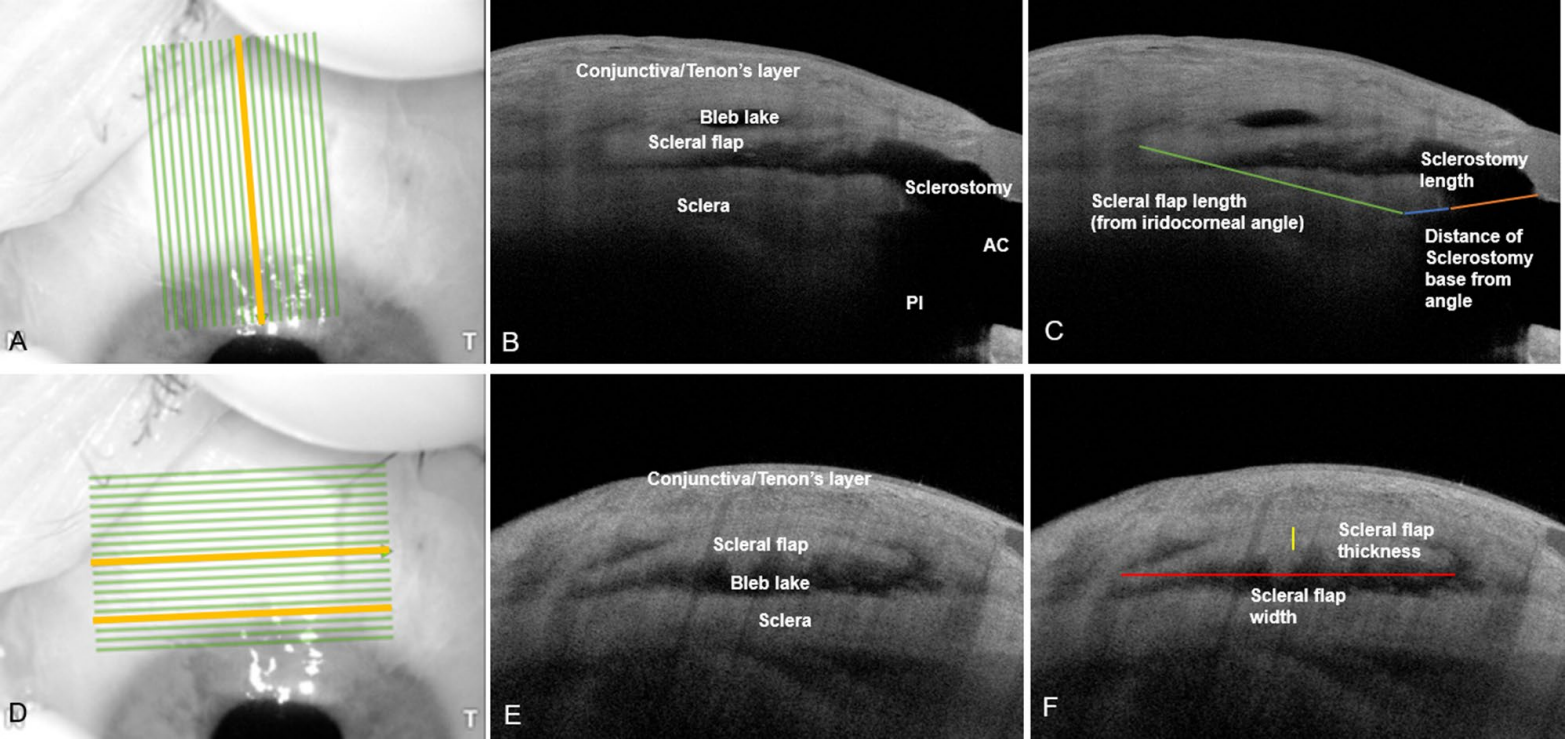
Enface (**A** and **D**) and sagittal AS-OCT images of a well-functioning trabeculectomy bleb with the overlying raster block shown in **A** and **D**, anatomical landmarks labelled in **B** and **E**, and surgical parameters of interest in **C** and **F**. The yellow line in panel A represents the sagittal slice overlying the sclerostomy and peripheral iridotomy, while the yellow lines in panel D represent the sagittal slice overlying the sclerostomy and mid-point of the scleral flap. Abbreviations: Anterior chamber (AC), Peripheral iridotomy (PI)

The trabeculectomy surgical site (filtering bleb) was first examined at the slit lamp by one of the authors (JT). AS-OCT of the filtering bleb was then performed by the same author using the Anterion ^®^ (Heidelberg Engineering GmbH, Heidelberg, Germany) swept-source OCT device. The Anterion^®^ uses a laser light source with a wavelength of 1300 nm and at 50,000 Hz to obtain B-scans with axial resolution of 10 microns and transverse resolution of 45 microns. Each scan was performed via the imaging module of the Anterion^®^ device using a standardised raster scan measuring 7.5 mm in width and 12 mm in length and comprising 19 slices. The raster block was first oriented parallel to the long axis of the scleral flap in the sagittal plane in relation to the bleb (Fig. 1A). The anterior limit of the image window was positioned just anterior to the limbus at the peripheral superior cornea, which allowed the sclerostomy, iridocorneal angle, and entire length of the scleral flap to be visualised and captured within the raster slices (Fig. 1B and C). The sagittal slice overlying the sclerostomy and peripheral iridotomy was identified from the 19 raster slices (yellow line in Fig. 1A) and exported for analysis. The raster block was then oriented perpendicular to the initial sagittal plane to visualise bleb structures in the coronal plane. The anterior limit of the raster block was placed anterior to the sclerostomy to allow the full width of the sclerostomy and scleral flap to be visualised and captured within the raster (Fig. 1E and F).

### Quantification of pixel intensity values and surgical dimensions

**Fig. 2:**
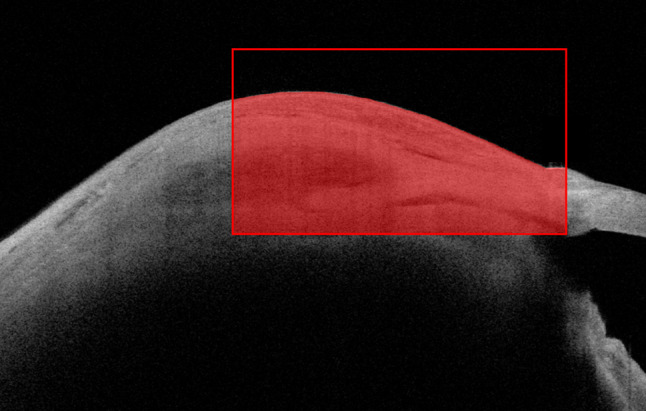
Standardised region of interest (ROI) measuring 400 by 800 pixels on the sagittal AS-OCT slice overlying the peripheral iridotomy and bisecting the scleral flap. This ROI is used for subsequent automated quantification of pixel intensity values

Bleb internal reflectivity was evaluated by measurement of pixel intensity values as follows: Standardised regions of interests measuring 400 × 800 pixels (px) capturing and bisecting the sclerostomy and scleral flap was cropped from sagittal AS-OCT images (Fig. 2). The following operations were performed using a custom written script in Matlab: [1] load original image [2], contrast adjust image followed by segmentation to create a binary mask of conjunctival/tenon’s layer and scleral flap based on pixel intensity values [3], filter largest pixel region from mask, which represents the bleb [4], measure the properties of the masked region obtained on the original image:

- Intensity-related pixel values:
  - Minimum intensity: value of pixel with lowest intensity.
  - Mean intensity: mean of all intensity values.
  - Maximum intensity: value of pixel with greatest intensity.
  - Solidity: proportion of pixels in convex hull, calculated as area/convex area [14].
  - Standard deviation of pixel intensity values.
- Area-related values.
  - Area of bleb: actual number of pixels in region.
  - Minor axis length (represents height of bleb).

Receiver operating characteristic (ROC) curves were used to examine the discriminatory ability of pixel intensity values to classify patients with surgical success (CS and QS) or failure.

Surgical parameters of interest were then quantified in accordance to standardised anatomical reference points (Table 1). The scleral flap length was measured using the iridocorneal angle as a reference point, as the anterior limit of the scleral flap was indistinct. The scleral flap length is therefore not a true flap length given the orientation of the plane of measurement, but rather served as a standardised measure from a well-defined anatomical landmark across different blebs.

**Table 1:** Surgical parameters of interest and associated standardised anatomical reference landmarks captured on the sagittal and coronal AS-OCT imaging of trabeculectomy blebs

Imaging plane	Surgical parameters of interest	Anatomical reference points of surgical parameter
Sagittal	Scleral flap length	Posterior edge of scleral flap to iridocorneal angle
	Sclerostomy length	Anterior edge of sclerostomy to posterior edge of sclerostomy
	Distance of Sclerostomy from iridocorneal angle	Posterior edge of sclerostomy to iridocorneal angle
Coronal	Scleral flap width	Nasal edge of scleral flap to temporal edge of scleral flap at midpoint of flap
	Scleral flap thickness	Superior edge of scleral flap to inferior edge of scleral flap at midpoint of flap
	Sclerostomy width	Nasal edge of sclerostomy to temporal edge of sclerostomy

Analyses were conducted using R (RStudio 2023.09.1 + 494) and GraphPad Prism (10.1.1).

## Results

100 filtering blebs of 65 patients were included in the study. The median post-operative follow-up duration was 7.0 years (IQR 3.2–16 years). The mean best-corrected visual acuity was 0.25 (Snellen equivalent of 20/36. SD 0.29) and visual field mean deviation was  -14.2 dB (SD 8.5) at the index clinic visit. The diagnoses were primary open angle glaucoma (79; 79%), pseudoexfoliation glaucoma (1;1%), primary angle closure glaucoma (12; 12%) and other/glaucoma not defined (8; 8%). 5 of the 100 blebs (5%) were second-site trabeculectomy surgeries.

### Correlation of bleb height and intensity values with surgical success

The proportion of complete success (CS; IOP ≤ 18mmHg with no medications), qualified success (QS; IOP ≤ 18 with medications) and failure (F; IOP > 18mmHg) was 45%, 33%, and 22% respectively. The mean intraocular pressure was 8.8 mmHg (SD 4.0), 13.2 mmHg (SD 3.1), and 16.3 mmHg (SD 10.6) in the complete success, qualified success and failure groups respectively. The mean number of medications was 0, 1.9 (SD 0.8) and 1.4 (SD 1.3) in the complete success, qualified success and failure groups respectively.

**Fig. 3:**
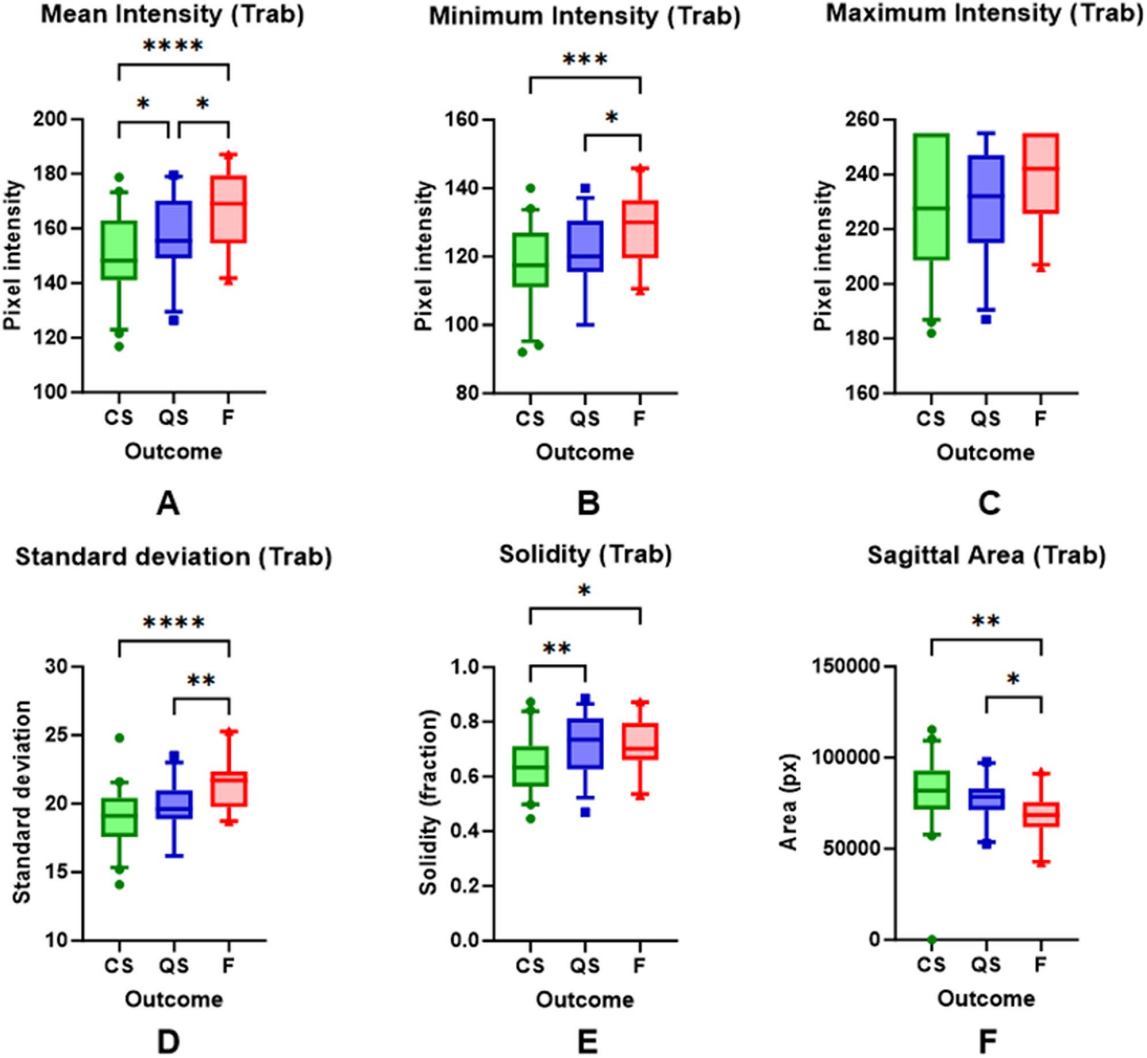
Box and Whisker plots (median, interquartile range and 5th to 95th percentile) and results of one-way ANOVA of bleb intensity values (mean, minimum, maximum, standard deviation and solidity of pixel intensity) and sagittal bleb area across the outcome groups of complete success (CS), qualified success (QS) and failure (F). Ns = non-significant. Asterisks denote statistical significance [ie. *p* < 0.05 (*), < 0.01 (**), < 0.001 (***), < 0.0001 (****)]

The mean maximum bleb height was significantly greater in the blebs with CS (1.74 vs. 1.25 vs. 1.23 mm in CS vs. QS vs. F, one-way ANOVA, *p* < 0.0001). Mean bleb area on sagittal section was significantly greater in blebs with CS (81.3 vs. 77.9 vs. 67.9k pixels, *p* = 0.0043, Fig. 3). Mean pixel intensity was significantly lower in blebs with complete success (150.8 vs. 157.4 vs. 167.4 in CS vs. QS vs. F, *p* = 0.0001). Other intensity values (minimum and maximum intensity, standard deviation and solidity) across the three outcome groups are shown in Fig. 3. We analysed the association between intensity values and success grouped by tertiles of post-operative period—early (1–4 years), middle (4–10 years) and late (beyond 10 years). Mean and minimum intensity remained significantly lower in CS compared to QS and F in the early post-operative phase (*P* = 0.009), but not in the middle- and late- post-operative phases. Standard deviation of intensity values was significantly lower in CS blebs in both the early- and middle- post-operative phases, but not in the late. There was also a significant association between mean, minimum and standard deviation of intensity values and success in cases of MMC use, but not in those without. These results are summarised in Table 2.

**Table 2:** Results of one-way ANOVA of intensity values (mean, minimum and standard deviation) against complete success, qualified success and failure grouped by post-operative phase and MMC use. Standard deviation of intensity values are presented in brackets. SD: standard. * denotes significance at the 5% level

	Complete Success	Qualified Success	Failure	*P* value
Post-operative phase				
*Early (0–4 years)*				
Mean	140.0 (13.4)	162.7 (12.8)	157.5 (11.0)	0.009*
Minimum	110.2 (11.2)	126.2 (10.6)	123.0 (7.9)	0.02*
SD	17.9 (2.2)	20.5 (1.6)	20.3 (1.0)	0.04*
*Middle (4–10 years)*				
Mean	153.0 (11.8)	157.2 (8.2)	166.0 (20.0)	0.17
Minimum	118.5 (8.7)	122.4 (6.7)	128.3 (15.3)	0.17
SD	19.0 (1.2)	20.2 (1.2)	21.4 (2.4)	0.01*
*Late (> 10 years)*				
Mean	150.7 (13.3)	153.6 (16.0)	168.2 (14.2)	0.22
Minimum	118.2 (9.5)	118.3 (12.1)	128.7 (10.1)	0.33
SD	19.2 (1.7)	19.1 (2.1)	20.8 (1.0)	0.35
**MMC use**				
Mean	148.2 (14.4)	157.9 (13.6)	170.2 (16.4)	0.005*
Minimum	115.8 (10.7)	122.7 (10.2)	131.6 (13.5)	0.008*
SD	18.7 (1.8)	20.1 (1.7)	21.6 (2.4)	0.002*
**No MMC use**				
Mean	151.7 (7.8)	152.6 (14.0)	166.2 (14.3)	0.17
Minimum	118.0 (7.4)	117.0 (11.1)	127.4 (9.9)	0.2
SD	19.1 (1.7)	18.7 (1.8)	21.0 (0.9)	0.06

### Discriminatory ability of pixel intensity values and bleb height in classifying success vs. failure

**Fig. 4:**
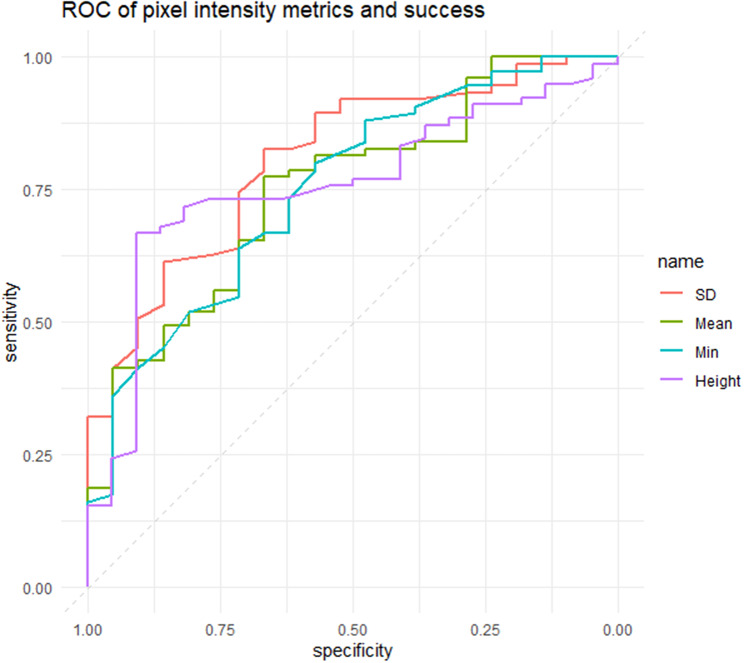
Receiver operating characteristic curves of standard deviation (SD) of pixel values, mean intensity, minimum intensity and bleb height to classify blebs with surgical success vs. failure

Receiver operating characteristic (ROC) curves were used to examine the discriminatory ability of pixel intensity values to classify patients with surgical success (CS and QS) or failure. AUC values for the pixel intensity values ranged from 0.63 to 0.81. Standard deviation of bleb intensity achieved the highest AUC of 0.81, followed by maximal height (AUC 0.76), minimum intensity (AUC 0.75), mean intensity (AUC 0.75) and minimum intensity (AUC 0.62). (Fig. 4)

### Correlation of scleral flap and sclerostomy dimensions with surgical success

**Fig. 5:**
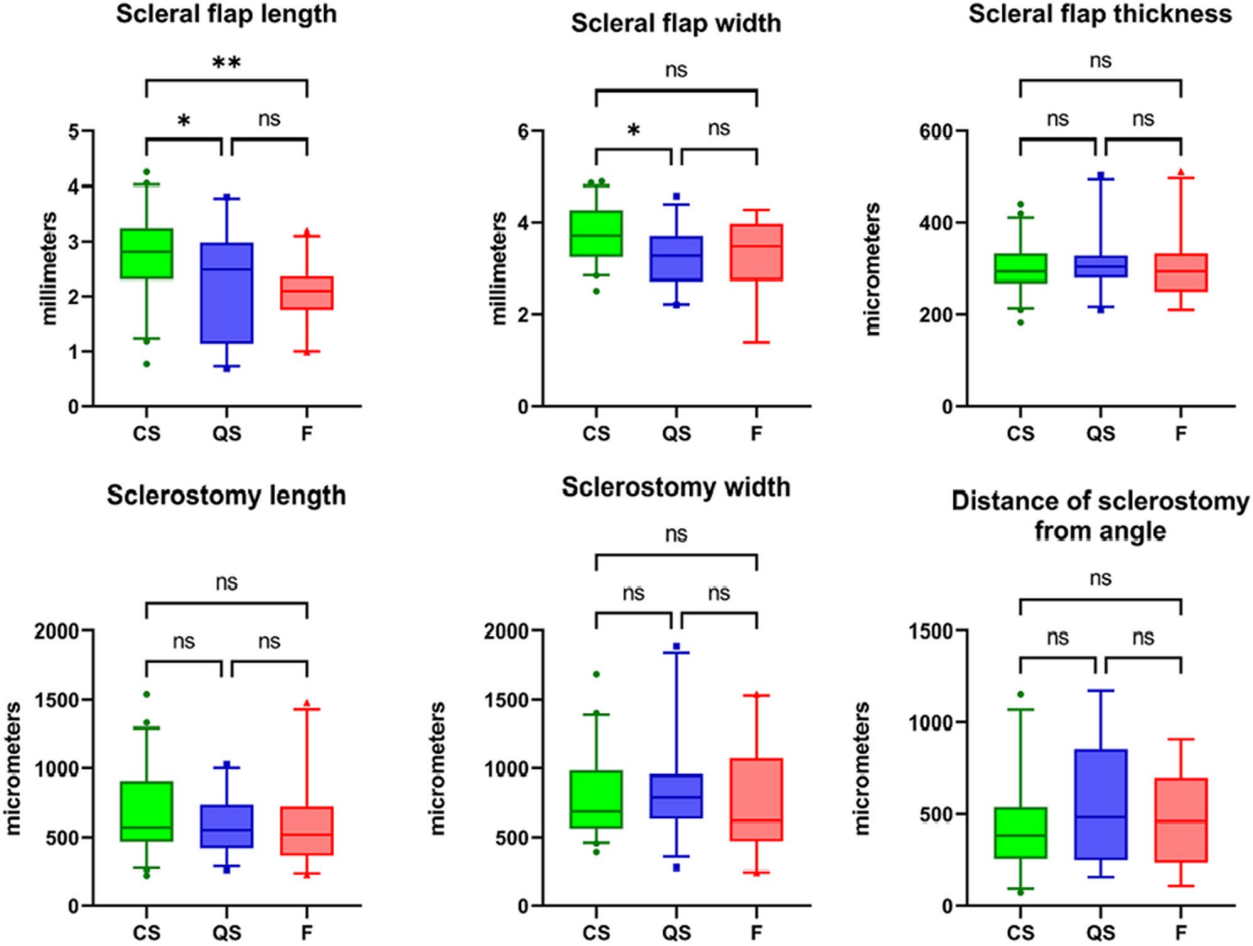
Box and Whisker plots (median, interquartile range and 5th to 95th percentile) and results of one-way ANOVA of scleral flap dimensions, sclerostomy dimensions and distance of the sclerostomy from the iridocorneal angle across the outcome groups of complete success (CS), qualified success (QS) and failure (F). Ns = non-significant. Asterisks denote statistical significance [ie. *p* < 0.05 (*), < 0.01 (**), < 0.001 (***), < 0.0001 (****)]

The scleral flap length as measured from the iridocorneal angle was significantly greater in the CS compared to the QS and F outcome groups. (2.76 vs. 2.15 vs. 2.08 mm in CS vs. QS vs. F, one-way ANOVA, *p* = 0.001). Scleral flap width was significantly greater in the CS compared to the QS group (3.76 vs. 3.24 vs. 3.25 mm in CS vs. QS vs. F, *p* = 0.003). Scleral flap thickness was similar across the three outcome groups. (299.8 vs. 311.3 vs. 302.5 μm in CS vs. QS vs. F, one-way ANOVA, *p* = 0.89). Sclerostomy length (665.5 vs. 579.6 vs. 590.5 μm in CS vs. QS vs. F, one-way ANOVA, *p* = 0.41) and sclerostomy width (776.3 vs. 860.0 vs. 741.3 μm in CS vs. QS vs. F, one-way ANOVA, *p* = 0.68) were also similar across the three outcome groups. The distance of sclerostomy from iridocorneal angle was smaller in the CS compared to QS and F groups, but this did not achieve statistical significance (421.2 vs. 545.2 vs. 485.1 μm in CS vs. QS vs. F, one-way ANOVA, *p* = 0.43). Figure 5 displays the dimensions of the surgical parameters of interest across the outcome groups of complete success, qualified success and failure.

## Discussion

This study demonstrates that anterior-segment optical coherence tomography can be successfully used to capture bleb internal reflectivity and morphology (height/area). While this has been previously published in the literature [9, 10, 15], this study demonstrates an objective method of quantifying these values in a standardized manner. Furthermore these values can be used to discriminate between well vs. poorly functioning blebs.

### Swept source AS-OCT in evaluating bleb morphology

The use of AS-OCT to examine filtering bleb morphology in glaucoma filtration surgery has been previously reported [16]. These studies have involved the use of spectral-domain OCT [9, 15], and have examined the association between intraocular pressure and internal bleb morphology, such as the height and thickness of the conjunctival/tenon’s layer [9, 15], bleb cavity [9, 10, 15], and presence of microcysts [11, 15], which reflect the aqueous dynamics induced by the drainage outflow channel. For instance, Lenzhofer et al. examined 78 eyes of 60 patients post-XEN^®^ gel stent implantation and found that the prevalence of small diffuse cysts was directly associated with lower IOPs, while cystic encapsulation at three months predicted higher surgical failure [17]. Konstantopoulos et al. examined 50 eyes of 50 patients following trabeculectomy, deep-sclerectomy or no surgery (controls), and found that a tall intrascleral lake and a thick conjunctival/tenon’s layer were associated with good post-operative outcomes as defined by intraocular pressure and medication use [15]. In recent years swept-source AS-OCT has enabled a more precise visualisation of these structures than preceding OCT modalities due to a greater penetration from longer wavelengths utilised and superior scan speeds [18]. Hasan et al. recently proposed a bleb classification system based on tomographical patterns at the conjunctival, tenons capsule and episcleral layer from 49 blebs following Xen gel implantation [19].

### Standardisation of location to capture bleb parameters

In this study, a standardised approach to the imaging location of the sagittal and coronal AS-OCT raster slices was implemented to maximise visualisation of the anatomical landmarks. Visualisation of internal bleb microstructure is heavily dependent on the location of the raster slice on the sclera [20]. Previous studies have found associations between internal bleb reflectivity and surgical success [11, 21, 22]. For example Narita et al. reported significant differences in the ratio of hypo-reflective space of the bleb wall in successful vs. unsuccessful blebs [21]. In this study, automated objective measurements of bleb intensity were obtained using a custom script on standardised sagittal sections. This is important in minimising bias from manual measurements. The mean global pixel intensity, minimum intensity, solidity and variance were significantly lower in complete success compared to qualified success and failure in the Trab blebs. (Fig. 3). Further longitudinal studies are required to determine if the mean intensity values measured change during the early post-operative period, where its use may guide pharmacological treatment/early surgical intervention. For instance, Kojima et al. measured bleb wall intensity longitudinally in 29 eyes following trabeculectomy, and found a decrease in intensity from 0 to 6 months post-operatively [22].

### Discriminatory ability of pixel intensity values and bleb height in classifying success vs. failure

**Fig. 6:**
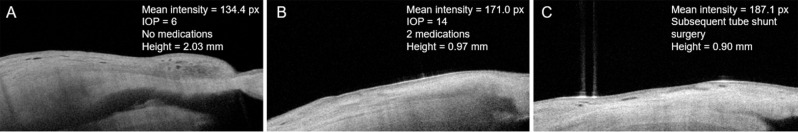
Representative blebs classified as complete success (A), qualified success (B) and failure (C) with associated global mean pixel intensity values, intraocular pressure (IOP), number of medications and maximal bleb height shown. Note the eye with bleb C underwent secondary tube shunt surgery, therefore bleb C was defined as having failed

We found moderate discriminatory ability of various pixel intensity values and bleb height in distinguishing between well- vs. poorly-functioning blebs, with surgical success at the 18mmHg threshold used as the ground truth. The highest AUC was achieved by standard deviation of pixel intensity values, followed by bleb height, mean intensity and minimum intensity. Standard deviation, or variance of pixel intensity values could be lower in well functioning blebs due to the homogenous architecture of underlying bleb tissues, which are often elevated and may display an aqueous lake and microcysts on sagittal section. [17]. Failed blebs in contrast often appear to be flatter with higher intensity values. (Fig. 6) Standardisation of post-operative follow up duration and controlling other potential confounders in the intra-operative and post-operative period may provide better quality data for the analysis, and allow these parameters to be investigated further. We nevertheless demonstrate a novel methodology of objectively classifying blebs with surgical success vs. failure using AS-OCT. Such a methodology may be helpful in complementing subjective bleb grading in routine clinical care. Further studies may be helpful in evaluating if these intensity values are dynamic and can reflect the degree of inflammation in the bleb tissues post-operatively, and the effect of pharmacotherapy in modifying tissue responses. We have previously demonstrated our methodology of bleb imaging in visualizing scleral flap patency, which was shown to be associated with success and MMC use [23], and which holds the potential of guiding post-operative bleb intervention such as needling. An objective, dynamic marker of bleb function using intensity values may therefore be helpful in guiding therapeutic interventions in the early post-operative period such as intensity of corticosteroid use.

### Limitations

We acknowledge several important limitations of our study. Firstly, as mentioned above there are other crucial determinants of surgical success which were not assessed in our analysis, predominantly due to the cross-sectional nature of our study of long follow-up duration. Some of these factors relate to pre-operative characteristics and surgical technique such as the intraoperative dimensions of the scleral flap, number and type of suture placement or the concentration and duration of intraoperative mitomycin-C use [24]. Postoperative care including suture and bleb manipulation can also affect surgical success [25]. Second-site trabeculectomy blebs may also have decreased success rates due to scarring associated with previous conjunctival incisional surgery. The primary focus of this study was however not to analyse the clinical outcomes of trabeculectomy surgery, but rather to optimise techniques for evaluating bleb reflectivity and morphology. Secondly, while quantification of bleb intensity values was performed using an automated script, the region of interest still had to be manually selected. Quantification of surgical parameters of interest also had to be performed manually due to the anatomical landmarks used to define boundaries, which may be subject to decreased reproducibility. We addressed this by having a second observer (MR) verify the measurements obtained. Computer vision algorithms may be potentially used to automate this process in future studies. Lastly, a threshold of 18mmHg and criterion of medication-use to classify surgical outcomes into complete success, qualified success, and failure in line with recommendations by the World Glaucoma Association consensus on definitions of success. Altering the IOP threshold may however change the distribution of our results. It’s crucial to acknowledge this, especially considering the established significance of pre-operative intraocular pressure, as well as the duration and quantity of prior topical medications, which are also linked to surgical outcomes. Considering these limitations a prospective study using AS-OCT to image trabeculectomy blebs in the post-operative period is required, where documentation of pre-operative parameters can strength the classification of success outcomes.

## Conclusion

Swept-source AS-OCT can be used to quantify bleb internal reflectivity and morphology, which can be used to distinguish between well vs. poorly functioning blebs. These parameters could assist surgeons in the objective evaluation of post-operative bleb outcomes.

## Data Availability

The datasets used and/or analysed during the current study are available from the corresponding author on reasonable request.

## Abbreviations

AS-OCT: Anterior segment Optical Coherence Tomography
Trab: Trabeculectomy
MMC: Mitomycin-C
CS: Complete success
QS: Qualified success
F: Failure
IOP: Intraocular pressure

## Declarations

### Meeting presentation

Presented at the Association for Research in Vision and Ophthalmology Annual meeting 2023, New Orleans, Louisiana.
